# Association of *Rhizoctonia theobromae* with cassava witches’ broom outbreak in Brazil and genetic relatedness to Southeast Asian isolates

**DOI:** 10.3389/fpls.2026.1799146

**Published:** 2026-05-28

**Authors:** Saulo Alves Santos de Oliveira, Samar Sheat, Paolo Margaria, Adilson Lopes Lima, Jackson de Araújo dos Santos, Hermínio Souza Rocha, Helton Fleck da Silveira, Cristiane Ramos de Jesus, Stephan Winter

**Affiliations:** 1 Embrapa Mandioca e Fruticultura, Comissão de Enfrentamento da Vassoura de Bruxa da Mandioca, Bahia, Brazil; 2 Deutsche Sammlung von Mikroorganismen und Zellkulturen (DSMZ), Plant Virus Department, Braunschweig, Niedersachsen, Germany; 3 Embrapa Pecuária Sul, Comissão de Enfrentamento da Vassoura de Bruxa da Mandioca, Bagé, Rio Grande do Sul, Brazil; 4 Embrapa Amapá, Macapá, Comissão de Enfrentamento da Vassoura de Bruxa da Mandioca, Amapá, Macapá, Brazil; 5 Embrapa Pesca e Aquicultura, Comissão de Enfrentamento da Vassoura de Bruxa da Mandioca, Palmas, Tocantins, Brazil

**Keywords:** Amazon region, cassava, *Ceratobasidium theobromae*, emerging plant disease, phylogenetics, quarantine pest, witches’ broom disease

## Abstract

**Background:**

A new cassava disease outbreak was identified in indigenous communities in Oiapoque, Amapá, Brazil, characterized by stunting, proliferation of thin shoots, broom-like leaf formations, and apical dieback. These symptoms are consistent with Cassava Witches’ Broom Disease (CWBD), previously reported in other regions of South America and Asia.

**Methods:**

Metagenomic profiling, molecular diagnostics, phylogenetic analyses, and multilocus genotyping were used to investigate microbial communities associated with symptomatic cassava plants.

**Results:**

Rhizoctonia (Ceratobasidium) theobromae was identified as the predominant fungal species associated with symptomatic plants. Genetic analyses indicated a close relationship between Brazilian isolates and Asian reference strains, suggesting a possible transcontinental introduction and supporting an association between R. theobromae and CWBD. This represents the first confirmed report of R. theobromae in Brazil, expanding its known geographic distribution in the Americas.

**Conclusion:**

The detection of this quarantine pathogen represents a potential threat to cassava production, food security, and preservation of indigenous cassava landraces in Brazil. These findings reinforce the need for surveillance, phytosanitary measures, and further studies on emerging fungal pathogens associated with cassava diseases.

## 1 Introduction

Cassava (*Manihot esculenta* Crantz) is the primary food source for approximately one billion people across 105 nations in the tropics and has substantial economic and social value, particularly in developing nations (Bayata, 2019; Muiruri et al., 2021). In recent years, it has been increasingly utilized as a source of bioenergy, starch, biopolymers, and beyond (Fathima et al., 2023). Brazil is the sixth largest producer of cassava tuberous roots in the world, with a production of 17.82 million tons (FAO, 2022), which is almost exclusively produced to satisfy domestic market demands. Thus, cassava is a vital source of food and income for Brazilians. A hitherto unknown disease is affecting cassava in the indigenous lands of the Oiapoque region in Brazil, bordering French Guiana, presenting a serious threat to the livelihoods of people relying on cassava as their main diet. The XXIX Assembly for the Evaluation and Planning of Indigenous People and Organizations in the municipality of Oiapoque, Amapá, Brazil convened in 2023 and requested urgent action to investigate the origin of the disease and provide solutions to mitigate its impact. Cassava grown in the forest exhibited abnormal growth with deformed leaves on the shoots. Short internodes and clustered leaves produce broom-like structures typical of cassava witches’ broom disease (CWBD). Leaf yellowing and vascular necrosis are further symptoms of the disease. Previously, similar symptoms have been observed in cassava grown in Southeast Asia (SEA) (Pardo et al., 2023) and recently, were also reported in French Guiana (Pardo et al., 2024). Eventually, the disease leads to the decline and wilting of leaves and stems, resulting in very thin and small roots.

A cassava witches’ broom-like disease was already reported in the 1940s from Brazil (Silberschmidt and Campos, 1944). Subsequent studies associated the so-called “oversprouting” symptoms with phytoplasmas belonging to the 16SrIII-B group (Previous studies associated cassava oversprouting in Brazil with 16SrIII-B phytoplasmas (Silberschmidt and Campos, 1944; Flôres et al., 2013). Oversprouting is characterized primarily by generalized shoot proliferation, chlorosis, and leaf deformation, without reports of consistent vascular necrosis. In contrast, the new cassava disease observed in Oiapoque is marked not only by shoot proliferation, but also by pronounced vascular necrosis, wilting, and progressive plant decline, ultimately leading to death. Cassava affected by CWBD in Southeast Asia (SEA), including Cambodia, Laos, Vietnam, Thailand, and the Philippines, was initially considered a phytoplasma-associated disease (Pardo et al., 2023). However, subsequent studies suggest the association of *Rhizoctonia theobromae* with the disease.

Using high-throughput sequencing (HTS), fungal isolation, and culture-based approaches, Leiva et al. (2023) and Gil-Ordóñez et al. (2024) identified R. theobromae (syn. Ceratobasidium theobromae) in symptomatic cassava plants from Southeast Asia. High-resolution microscopy revealed fungal colonization of xylem tissues associated with vascular necrosis (Gil-Ordóñez et al., 2024), while transmission through wedge grafting further supported its association with CWBD (Leiva et al., 2023). Similarly, Landicho et al. (2024), using PCR and HTS, reported that microbial communities in CWBD-affected cassava from the Philippines were dominated by *R. theobromae* sequences.

Our research on CWBD in cassava from Oiapoque was guided by these previous findings. To investigate the microbial communities associated with diseased cassava, we employed a high-throughput sequencing (HTS) approach for metagenomic analysis. A large portion of the DNA sequences not mapped to the cassava genome corresponded to *R. theobromae*, although sequences from other microorganisms were also detected. In addition, genetic analyses based on Ca2+/calmodulin-dependent protein kinase (CAMK/CAMKL) sequences were performed to compare Brazilian isolates with reference isolates from other geographic regions. The CAMK/CAMKL locus was selected because it has been previously validated for species-specific detection of *R. theobromae* (Leiva et al., 2023; Gil-Ordóñez et al., 2024) and represents one of the few loci currently available in public repositories for comparative analyses across geographic regions.

## 2 Materials and methods

**Figure 1:**
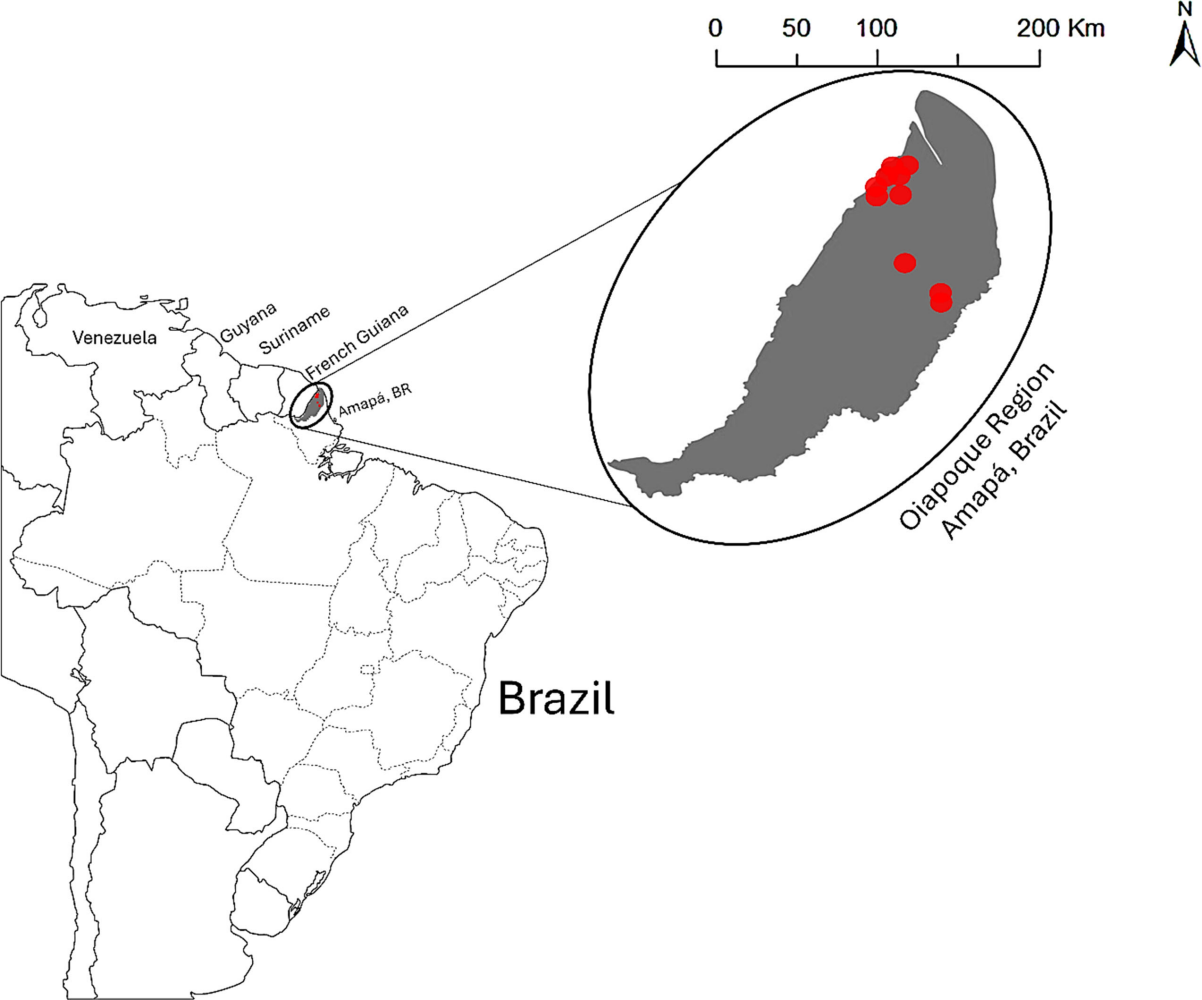
Collection of cassava samples from diseased plants in Kuai Kuai, Ariramba, Galibi, Lençol, Ahumã, Anawerá, and Yanawaká. Red dots indicate collection sites.

Fifty samples were collected from cassava fields in Kuai Kuai, Ariramba, Galibi, Lençol, Ahumã, Anawerá and Yanawaká, in the Oiapoque Region, Amapá, Brazil (**Figure 1**). Cassava fields in the indigenous territories of Amapá are established within small forest clearings locally known as “roça de toco”. Plants are distributed irregularly according to traditional agricultural practices. Because of this irregular planting pattern, structured transect-based sampling was not feasible. Therefore, a cross-sectional sampling strategy was adopted, collecting symptomatic and asymptomatic plants opportunistically across each field. Field sizes ranged from 0.1 to 1.2 ha.

Sampling was conducted between September 18–23, 2023, with additional collections conducted during the subsequent rainy season in 2024. Fields consisted of mixed local landraces, with 3–5 cassava varieties cultivated within the same field. According to reports from indigenous farmers, prior to the outbreak, up to 68 distinct local genotypes were traditionally cultivated within the same community areas. At the time of sampling, plants ranged from 4–8 months of age.

Leaves, petioles, and stems from symptomatic cassava plants were collected (**Table 1**) Following SISGEN registration (AFB8348), samples were packaged in sealed containers and transported to Embrapa Mandioca e Fruticultura in Cruz das Almas, Bahia, Brazil.

**Table 1:** Overview of samples collected from different villages in 2023.

Pool	Sample n°	Symptoms	Village	Tissue	Collection year
Asymptomatic	2657	none	Ahumã	Leaf	2023
Asymptomatic	2205	none	Ariramba	Leaf	2023
Asymptomatic	2411	none	Lençol	Leaf	2023
Asymptomatic	2703	none	Anawerá	Leaf	2023
Asymptomatic	2805	none	Yanawaka	Leaf	2023
DWBP	2307	Witches’ broom	Galibi	Petiole	2023
DWBP	2610	Witches’ broom	Ahumã	Petiole	2023
DWBP	2704	Witches’ broom	Anawerá	Petiole	2023
DWBP	2403	Witches’ broom	Lençol	Petiole	2023
DWBP	2809	Witches’ broom	Yanawaka	Petiole	2023
DWBS	2309	Vascular necrosis	Galibi	Stem	2023
DWBS	2401	Vascular necrosis	Lençol	Stem	2023
DWBS	2606	Vascular necrosis	Ahumã	Stem	2023
DWBS	2702	Vascular necrosis	Anawerá	Stem	2023
DWBS	2207	Vascular necrosis	Ariramba	Stem	2023

Asymptomatic, no visible symptoms.

DWBP, Pool of samples containing total DNA extracted from the petioles of plants exhibiting witches’ broom symptoms.

DWBS, Pool of samples containing total DNA extracted from the stems of plants displaying both witches’ broom symptoms and vascular necrosis.

Freeze-dried samples were stored at -80 °C prior to shipment to the Leibniz Institute (DSMZ, Germany).

### 2.1 Pathogen detection based on high throughput sequencing

HTS sequencing and subsequent bioinformatics analyses were conducted at the Plant Virus Department (Leibniz Institute DSMZ, Braunschweig, Germany). Total DNA was extracted from each sample (150 mg of stem, leaf, or petiole tissue) using the NucleoSpin^®^ Plant II kit (MACHEREY-NAGEL GmbH & Co. KG, Germany), following the manufacturer’s instructions. DNA quality and concentration were assessed using a NanoDrop 2000 spectrophotometer (Thermo Fisher Scientific, USA). DNA quantification for library preparation was performed using the Qubit™ dsDNA High Sensitivity (HS) Assay Kit on a Qubit 4 Fluorometer (Thermo Fisher Scientific, USA).

HTS of total DNA from cassava was performed using tissue-specific sample pools (leaves, petioles, and stems). Equal amounts of DNA were pooled consisting of DNA from non-symptomatic leaves, petioles from witches’ broom plants, and stems with vascular necrosis. Library preparation was performed using the Illumina DNA Prep Kit (FlexM; Illumina Inc., USA) with IDT^®^ for Illumina^®^ DNA/RNA UD Indexes Set D (Integrated DNA Technologies, USA).

Double-stranded cDNA (ds-cDNA) was generated using commercially available kits and enzymes for rRNA depletion, reverse transcription, second-strand synthesis, and purification, following manufacturers’ instructions.

Sequencing was conducted on a NextSeq 2000 instrument using paired-end 2 × 150 bp reads. Illumina reads were quality trimmed using BBDuk (BBTools suite) to remove adapters and low-quality bases. Coverage normalization was performed using BBNorm (BBTools suite), to reduce redundancy prior to assembly. Duplicate reads were removed using Dedupe (BBTools suite). Processed Illumina reads were mapped against the *M. esculenta* reference genome (NCBI RefSeq assembly GCF_001659605.2, Annotation Release 101) using BBMap (BBTools suite) implemented in Geneious Prime (v2024.0; Biomatters), with unmapped reads retained for *de novo* assembly. *De novo* assemblies were performed in Geneious Prime using both the ‘Geneious *de novo* assembler’ and the SPAdes assembler plugin. Hybrid were generated using SPAdes within Geneious Prime.

ONT libraries were prepared using the Rapid PCR Barcoding Kit (SQK-RPB004; Oxford Nanopore Technologies, UK) and sequenced on a MinION Mk1C device using R9.4.1 flow cells (FLO-MIN106). Raw reads were base called and demultiplexed using Guppy (v6.0.1). Adapter trimming and read filtering were performed using Porechop Processed ONT reads were mapped against the *M. esculenta* reference genome (NCBI RefSeq assembly GCF_001659605.2) using Minimap2 v2.26 (Li, 2018), with unmapped reads retained for downstream analyses.

### 2.2 Bioinformatic analysis

For taxonomic profiling, *de novo* assembled contigs from Illumina and ONT reads were analyzed using DIAMOND-BLASTx v2.1.11 (Buchfink et al., 2015) against the NCBI non-redundant (nr) protein database (downloaded January 10, 2024). The database was supplemented with curated fungal pathogen protein sequences from the DSMZ collection that were not yet publicly available. Taxonomic assignments were performed during DIAMOND alignment. The resulting output was imported into MEGAN6 (Huson et al., 2016) for visualization and summarization.

For additional taxonomic support, trimmed reads and assembled contigs were also screened against a custom database of plant-associated fungal reference sequences using BLASTn/BLASTx. This complementary analysis was used to improve confidence in species-level identification.

Partial genome scaffolds obtained from Illumina and ONT hybrid assemblies were compared with those of closely related organisms for comparative taxonomic analyses. Pairwise comparisons were conducted using scaffolds from DWBP and DWBS samples, *Rhizoctonia solani* AG-1 IA (GCF_016906535.1), and *Rhizoctonia theobromae* LAO01 (GCA_037974915.1). Additional analyses were performed using taxonomically informative loci, including large subunit ribosomal RNA (LSU), translation elongation factor 1-alpha (tef1), RNA polymerase II subunit (rpb2), and ATP synthase subunit 6 (atp6), for comparison with reference genomes of *R. theobromae* strains (GCA_037974915.1, GCA_009078325.1, GCA_012932095.1).

### 2.3 Phylogenetic analyses

ITS rDNA regions (ITS1, 5.8S, and ITS2) from fungi associated with CWBD-affected cassava were aligned with reference isolates from various species complexes using the MUSCLE algorithm in MEGA v11 (Tamura et al., 2021). Reference sequences were selected from GenBank based on sequence similarity. *Scotomyces subviolaceus* and *Heteroacanthella acanthophysa* were used as outgroups. Phylogenetic analysis was conducted using the Maximum Likelihood (ML) method in MEGA v11 with 1000 bootstrap replicates. Pairwise nucleotide identities were calculated using T92+G substitution model selected based on BIC and AIC criteria. Trees were visualized and edited using FigTree v.1.4.4 (available at http://tree.bio.ed.ac.uk/software/figtree).

Multilocus phylogenetic analysis (MLG) was performed using concatenated sequences of the ITS region (partial 18S, ITS1, 5.8S, ITS2, and partial 28S), LSU (28S), and mitochondrial atp6 genes. This analysis included isolates of *Rhizoctonia theobromae* and *Rhizoctonia solani*. A second phylogenetic tree was constructed using ITS and LSU regions to include additional Asian *R. theobromae* isolates infecting cacao (*Theobroma cacao*) and cassava.

Pairwise nucleotide identity were calculated using the T92+G model selected using on BIC and AIC criteria. Branch support was additionally assessed using both Neighbor-Joining (NJ) and Maximum Parsimony (MP) performed alongside the MLG analyses. Trees were visualized and edited using FigTree v1.4.4.

### 2.4 PCR-based detection

Detection of *R. theobromae* was performed using the primers CWBD-CIAT-F2 (GGATGAGTTTAATCGCTCTAAC) and CWBD-CIAT-R2 (GCGCTCTGGTGTTTCAAGTTTG) (Leiva et al., 2023), targeting the putative Ca2+/calmodulin-dependent protein kinase gene (CAMK/CAMKL) (Leiva et al., 2023). PCR reactions were performed in 25 µL volumes containing standard concentrations of buffer, primers, MgCl_2_, dNTPs, Taq polymerase, and template DNA. PCR amplification consisted of 35 cycles with denaturation at 95 °C, annealing at 52 °C, and extension at 72 °C, and after that subjected to bidirectional Sanger sequencing. Leaves, petioles, and stems were analyzed to account for possible differences in pathogen detection among tissues. To assess assay specificity, plants displaying symptoms of anthracnose, frogskin disease, and oversprouting were collected from the Embrapa Cassava Germplasm Bank in Cruz das Almas, Bahia, Brazil, and tested using CAMK/CAMKL primers.

#### 2.4.1 *Rhizoctonia theobromae* genetic diversity analysis

CAMK/CAMKL sequences obtained from Sanger sequencing were quality-checked, assembled, and manually edited to generate consensus sequences for each isolate. Given the limited availability of multilocus datasets for *R. theobromae*, this locus was used as a preliminary marker for comparative genetic diversity analyses. The consensus sequences were compared with those available in GenBank using BLASTn (National Center for Biotechnology Information, 2024) (cited 2024 Oct 02). Available from: https://www.ncbi.nlm.nih.gov.

The generated consensus sequences were aligned using the MUSCLE algorithm implemented in MEGA v11 together with Asian CWBD-associated isolates. Sequences were manually trimmed to a uniform length of 738 bp prior to haplotype analyses. Terminal regions containing ambiguous bases (Ns) and low-quality sites were removed to ensure improve quality, only high-confidence positions were retained for downstream analyses.

DnaSP ver. 6.12.03 (Rozas et al., 2017) was used to calculate the genetic diversity indices. Haplotype diversity (Hd) and nucleotide diversity (π) were calculated. Relationships among were evaluated using a haplotype network based on single nucleotide variation (SNV) constructed using the median-joining method using PopART 1.7 software (Leigh and Bryant, 2015). Nexus files generated in DnaSP were used as the input for PopART 1.7. Haplotype were colored according to country of origin of the *R. theobromae* CAMK/CAMKL DNA sequences.

## 3 Results

**Figure 2:**
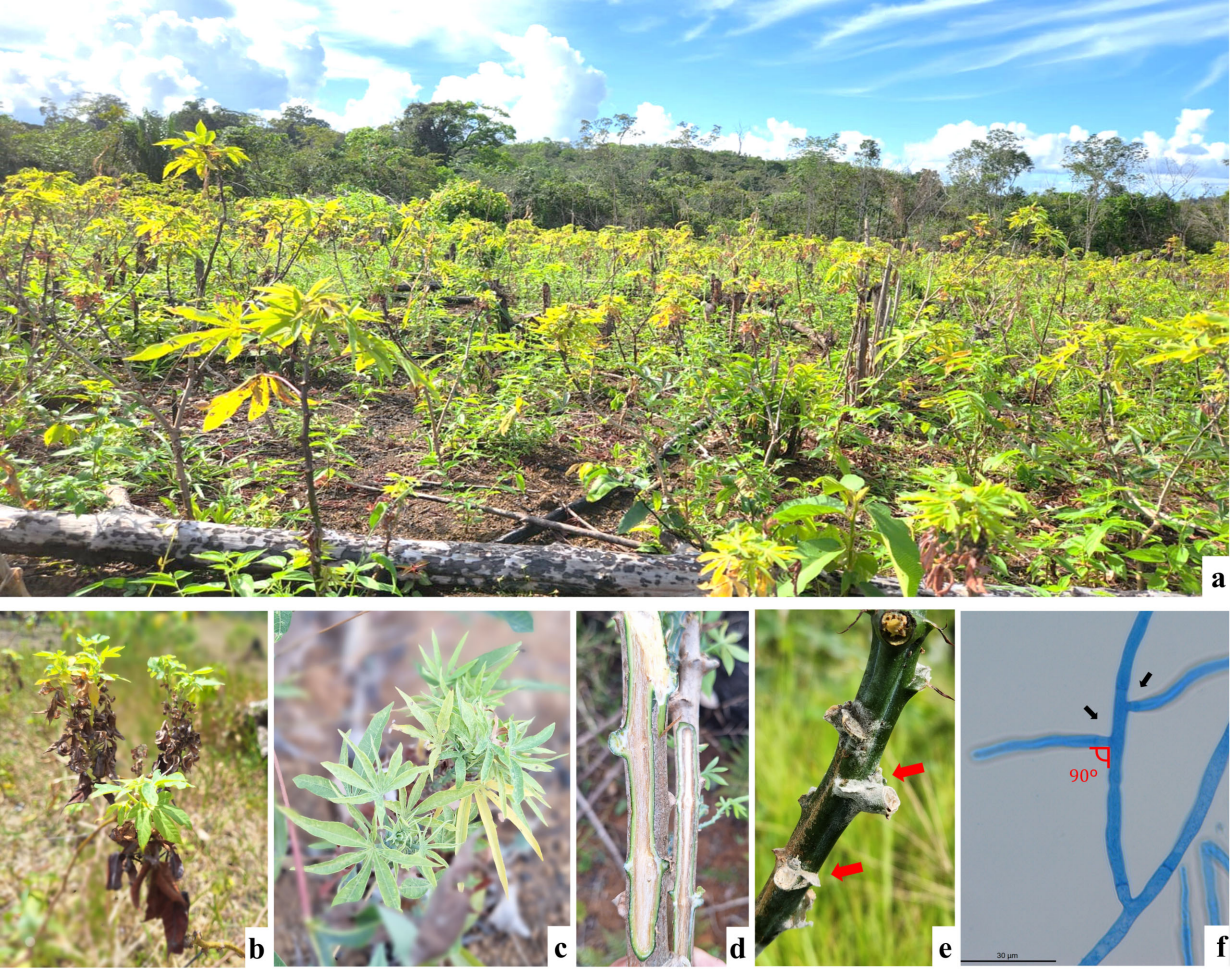
Aspects of cassava plants with symptoms of “oversprouting” associated with a 16SrIII-B phytoplasma. Plants showing abnormal “bushy” shoot emissions and stunting (dwarfism) of young plants **(a–c, e)**. Longitudinal section of a cassava stem showing healthy vascular tissue without necrosis **(d)**; whitish mycelium oozing out of nodes indicating fungal infection **(e)**; and typical “*Rhizoctonia*-like” hyphae isolated from petioles, stained with lactophenol blue, and observed under a light microscope (×400) **(f)**.

Characteristic symptoms of CWBD were observed in cassava from indigenous villages in the Oiapoque Region, Amapá, Brazil (**Figures 2a–c**). Symptoms included stunting and proliferation of short, thin and weak shoots on cassava stems, resulting in the formation of “brooms”, represented by short internodes, aggregated rosette-shaped leaves aggregation and vascular necrosis. In severe cases, a cotton-like mycelium oozed out of the petiole base near the buds (**Figure 2e**). The fungus produced characteristic aerial light-yellowish mycelia with hyphal branches at approximately 90° angles (**Figure 2e**). These branches exhibited constrictions at their bases, immediately followed by septa beyond the branching point (**Figure 2f**). As the disease progresses, chlorosis, wilting and drying of leaves, apical death, and descending plant death occur.

Fungal isolates obtained from symptomatic cassava petioles produced slow-growing colonies on 2% malt extract agar, initially white and becoming light cream with age. Microscopic examination revealed septate hyphae with right-angle branching typical of *Rhizoctonia*-like fungi (**Figure 2f**). However, subculturing proved difficult, as isolates frequently exhibited reduced growth after successive transfers and rapidly lost viability *in vitro*. In several cases, cultures became senescent or failed to regrow after storage, limiting long-term maintenance. These morphological and cultural characteristics, together with the molecular data, supported the identification of the isolates as *R. theobromae*-like fungi, consistent with molecular data.

Three fungal isolates were initially obtained from symptomatic cassava petioles; however, due to limited stability and rapid loss of viability in culture, only one isolate remained viable for pathogenicity assays. This isolate was inoculated onto cassava plantlets under greenhouse and *in vitro* conditions. Inoculated plants developed localized necrotic lesions at the inoculation site, and in some cases rapid plant decline and death were observed. However, the characteristic witches’ broom phenotype, including rosette proliferation and shortened internodes, was not reproduced under controlled conditions. However, the inability to reproduce the characteristic witches’ broom phenotype indicates that the role of this fungus in the full disease expression remains unresolved.

### 3.1 CWBD and “oversprouting” disease

**Figure 3:**
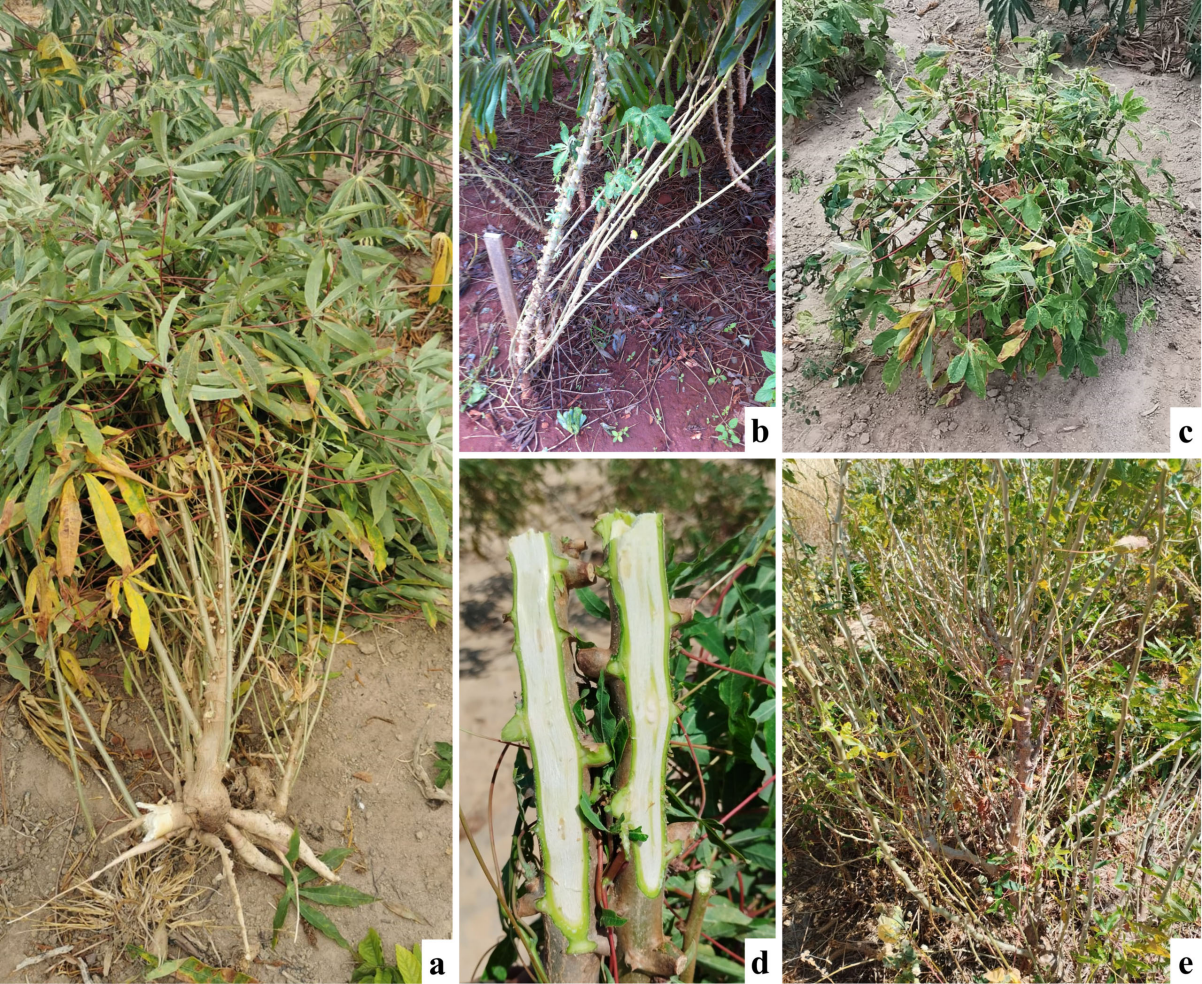
Aspects of cassava plants with symptoms of “Oversprouting” caused by a 16SrIII-B phytoplasma. Plants showing abnormal “bushy” shoot emissions and stunting (dwarfism) of young plants **(a–c, e)**. Longitudinal section of a cassava stem with a healthy appearance and intact vascular tissue **(d)**.

This new disease of cassava, CWBD, shares similarities with the phytoplasma-associated disease “superbrotamento” or “Brazilian witches’ broom”, herein referred to as ‘Oversprouting’. Both result in abnormal shoot proliferation and stunted plants, but “Oversprouting” is characterized by generalized stunting, excessive shoot proliferation, and leaves that are chlorotic, deformed, and smaller than normal. The new stems are unusually thin and produced in large numbers (**Figure 3**).

In contrast to oversprouting (phytoplasma-associated), CWBD is characterized not only by dwarfism and shoot proliferation, but also by the formation of well-defined broom-like structures with markedly shortened internodes and rosette-shaped leaf clusters. A distinctive feature of CWBD is the consistent presence of vascular necrosis in affected stems, which is not observed in oversprouting. As CWBD progresses, vascular discoloration leads to wilting, apical dieback, and eventual plant death.

Molecular analyses confirmed that plants displaying oversprouting symptoms were associated exclusively with phytoplasma infection and not with *Rhizoctonia*. PCR amplification of the 16S rRNA gene followed by Sanger sequencing identified phytoplasma strains belonging to the 16SrIII-B subgroup, consistent with previous reports for cassava oversprouting disease (Flôres et al., 2013; Flores et al., 2023). Representative sequences obtained in this study were deposited in GenBank under accession numbers PX515190 and PX515191. Importantly, no *Rhizoctonia* sequences were detected in oversprouting samples, reinforcing that cassava oversprouting and cassava witches’ broom disease represent distinct disease entities with different etiological agents.

### 3.2 HTS sequencing for detection of pathogens

Since the symptoms observed in cassava plants closely resembled those previously described for witches’ broom disease in Asia (Leiva et al., 2023; Gil-Ordóñez et al., 2024) and recently reported in French Guiana (Pardo et al., 2024) the presence of *Rhizoctonia theobromae* (syn. *Ceratobasidium theobromae*) was investigated. To investigate potential microbial agents associated with CWBD in Brazil, Illumina sequencing was performed on pooled samples from asymptomatic plants, plants exhibiting witches’ brooms (DWBP), and plants showing vascular necrosis (DWBS) (**Table 1**).

Approximately 84.91% of the reads from the asymptomatic pool mapped to the cassava genome, whereas mapping rates were lower in symptomatic plants, with 78.78% in the DWBS pool and 70.76% in the DWBP pool. Notably, *Rhizoctonia theobromae* contigs were detected exclusively in the DWBP and DWBS pools in this study. The median number of reads obtained per pool ranged from 48, 823, 878 to 62, 648, 108, with 500 and 495 contigs identified in DWBP and DWBS, respectively (**Table 2**).

**Table 2:** Results from Illumina sequencing after quality filtering.

Illumina data	Samples		
	Asymptomatic	DWBP	DWBS
Total reads	62, 648, 108	48, 823, 878	60, 609, 962
Total reads after normalization	29, 673, 340	29, 698, 224	33, 353, 050
% Reads mapped to cassava	84, 91%	70, 76%	78, 78%
# Reads unmapped	4, 476, 529	8, 684, 988	7, 076, 949
Number of produced contigs	6, 020	5, 250	4, 975
Minimum Length (bp)	489	431	357
Maximum Length (bp)	42, 266	53, 407	32, 731
*Rhizoctonia theobromae* contigs	0	500	495

Asymptomatic, DNA from a pool of asymptomatic leaves.

DWBP, DNA from petiole of plants expressing clear symptoms of cassava witches’ broom disease.

DWBS, DNA from stem of plants expressing clear symptoms of cassava witches’ broom disease and with vascular necrosis.

Total reads, Number of total reads obtained after quality check, filtering and trimming.

# Reads unmapped, Number of reads not belonging to the host.

*Rhizoctonia theobromae* contigs, Number of contigs assigned to *Rhizoctonia* and *Ceratobasidium* genomes.

ONT sequencing generated long-read data that corroborated the Illumina-based taxonomic assignments. No additional fungal taxa or major sequence variants were detected exclusively in the ONT dataset. Therefore, Illumina and ONT reads were combined for hybrid assembly to improve contig continuity and genome reconstruction.

### 3.3 Phylogenetic analyses

Previous research has shown that the *genus Ceratobasidium* is associated with witches’ broom disease. Hence, *Rhizoctonia* sp. sequences from the ITS rDNA region, including partial sequences of the 18S and 28S rDNA genes, internal transcribed spacers 1 and 2 (ITS1 and ITS2), and the 5.8S rDNA gene were analyzed. Sequences from HTS were mapped against a randomly chosen *Rhizoctonia solani* reference sequence (GenBank accession no. AF153800). The resulting consensus sequences were used for subsequent phylogenetic analyses. In the alignment matrices, sequences from the first report of *Ceratobasidium* sp. associated with cassava in Asia (Leiva et al., 2023) were used as reference sequences (**Table 3**).

**Table 3:** Description of the isolates used for phylogenetic analysis based on the ITS rDNA region of *Rhizoctonia-like* species, with collection details and GenBank accession numbers.

GenBank	Taxonomic assignation	Location	Host	Isolation source
AB122145	*Ceratobasidium* sp. AG-k	Japan	*Allium cepa*	mycelium
AB196647	*Ceratobasidium* sp.	Japan	*Arachis hypogaea*	mycelium
AB196650	*Ceratobasidium* sp. AG-I	Japan	*Artemisia* sp.	mycelia
AJ427407	*Ceratobasidium* sp. AGR	USA	*Cucumis* sp.	mycelia
AF354095	*Ceratobasidium* sp. AGQ	Japan	Cynodon_Soil	mycelia
KY075643	*Ceratobasidium theobromae*	China	*Lonicera japonica*	--
KP119764	*Ceratobasidium theobromae*	China	*Lonicera japonica*	--
KP119766	*Ceratobasidium theobromae*	China	*Lonicera japonica*	--
KP119765	*Ceratobasidium theobromae*	China	*Lonicera japonica*	--
OR145521	*Ceratobasidium theobromae*	Cambodia	*Manihot esculenta*	petiole
OR145522	*Ceratobasidium theobromae*	Vietnam	*Manihot esculenta*	petiole
OR145523	*Ceratobasidium theobromae*	Lao PDR	*Manihot esculenta*	petiole
AB286940	*Ceratobasidium* sp. AG-P	Japan	--	--
AB290017	*Ceratobasidium* sp. AG-E	Japan	--	--
AB000019	*Rhizoctonia solani*	Japan	--	--
AY154313	*Thanatephorus cucumeris*	Brazil	--	--
AF354108	*Thanatephorus cucumeris*	Veracruz	*Solanum tuberosum*	mycelia
AF153804	*Rhizoctonia solani*	Australia	*Pterostylis acuminata*	--
AB286930	*Ceratobasidium* sp. AG-Ba	Japan	Rice	--
AB196659	*Ceratobasidium* sp. AG-T	Japan	*Rosa odorata*	--
AB196653	*Ceratobasidium* sp. AG-L	Japan	Soil	mycelium
AB000003	*Rhizoctonia solani*	Japan	Soil	--
AF153800	*Rhizoctonia solani*	Australia	Soil	--
AB196649	*Ceratobasidium* sp. AG-H	Japan	Soil	mycelia
AB290021	*Ceratobasidium* sp. AG-C	Japan	Sugar beet	mycelia
KU255726	*Ceratobasidium theobromae*	Indonesia	*Theobroma cacao*	leaf
KU319572	*Ceratobasidium theobromae*	Indonesia	*Theobroma cacao*	leaf
KU255724	*Ceratobasidium theobromae*	Indonesia	*Theobroma cacao*	petiole
KM523668	*Ceratobasidium theobromae*	Indonesia	*Theobroma cacao*	mycelium
KU319573	*Ceratobasidium theobromae*	Indonesia	*Theobroma cacao*	leaf
KU310940	*Ceratobasidium theobromae*	India	*Theobroma cacao*	petiole
KU310941	*Ceratobasidium theobromae*	India	*Theobroma cacao*	petiole
AB000004	*Rhizoctonia solani*	U.S.A.	Tobacco	--
AB198702	*Ceratobasidium* sp. AG-D	Japan	*Zoysia japonica*	mycelia
MZ159527	*Scotomyces subviolaceus*	Scotland	--	--
MT340869	*Heteroacanthella acanthophysa*	--	--	--
PQ192611	*Ceratobasidium theobromae*	Brazil	*Manihot esculenta*	petiole
PQ192612	*Ceratobasidium theobromae*	Brazil	*Manihot esculenta*	stem

**Figure 4:**
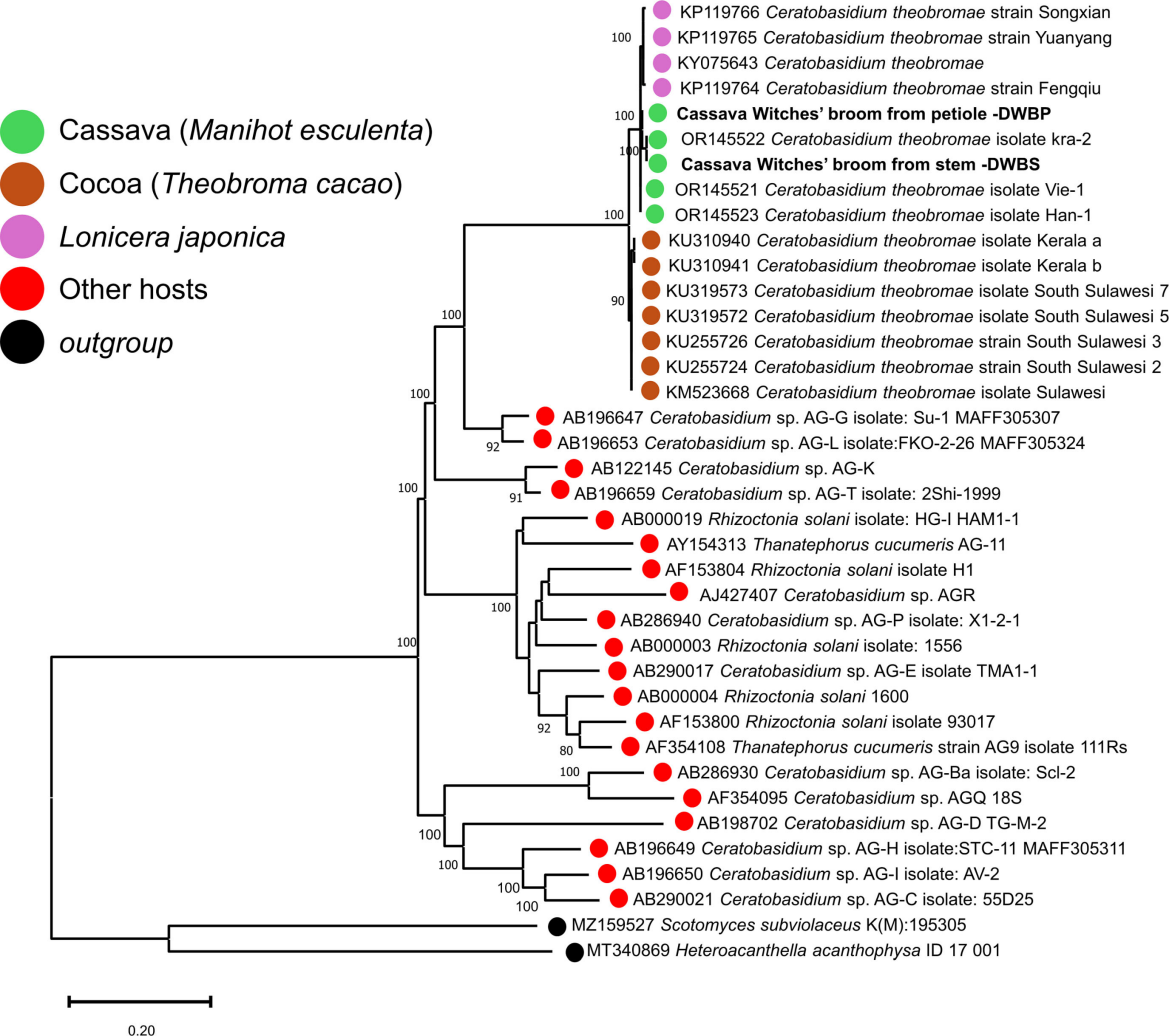
Relationship between *Rhizoctonia theobromae* from cassava collected from indigenous villages in the Brazilian rainforest in the Oiapoque region and others ‘*Rhizoctonia*-like*’* species sequences available in GenBank based on the internal transcribed spacer (ITS) sequence of the rDNA. A set of isolates found causing vascular necrosis in cacao (*Theobroma cacao*) and honeysuckle (*Lonicera japonica*) and witches’ broom in cassava (*M. esculenta*) in Asian countries were also included. The tree was constructed using the maximum-likelihood method based on the Tamura 3-parameter with gamma distribution (T92+G) model with 1000 bootstrap replicates. Bootstrap values higher than 70% are displayed near the branch. *Scotomyces subviolaceus* and *Heteroacanthella acanthophysa* were used as outgroup.

The two ITS rDNA consensus sequences assembled from the DWBS and DWBP samples were placed in a well-supported clade comprising only *C. theobromae* (formerly *R. theobromae*) (**Figure 4**). The subclade formed assigned isolates from cassava and cacao associated with witches’ broom disease (CWB) and vascular streak dieback (VSD), respectively.

A multilocus phylogenetic analysis (MLG) was conducted to assess the molecular relationships of *Rhizoctonia theobromae* isolates from Brazil. Sequence identity comparisons with the *C. theobromae* LAO1 reference genome (GCA_037974915.1) revealed 100% identity for the *atp6* gene, 98.47% for the ITS rDNA region, and 100% for LSU, indicating a high degree of genetic similarity.

**Figure 5:**
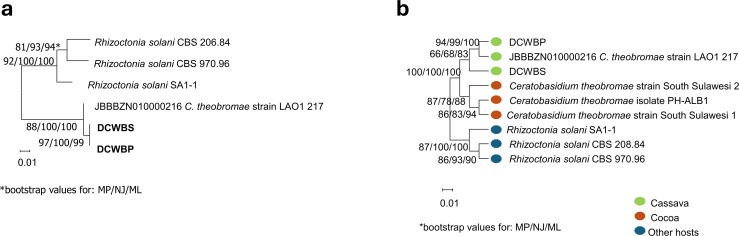
Phylogenetic analysis of *Rhizoctonia theobromae* isolates was performed using multilocus phylogenetic analysis (MLG). **(a)** MLGs using Brazilian *R. theobromae* (syn. *Cb. theobromae*) isolates and additional *R. solani* isolates based on the ITS, LSU, and *atp6* regions. **(b)** MLGs using ITS + LSU regions with additional isolates from cassava and cacao. Colored circles indicate the origin of the isolates.

The MLGs based on ITS, LSU, and *atp6* regions consistently resolved *R. theobromae* isolates into a distinct clade from *R. solani*, with strong bootstrap support of 88%, 100%, and 100% for Maximum Parsimony (MP), Neighbor-Joining (NJ), and Maximum Likelihood (ML), respectively (**Figure 5a**). When additional *R. theobromae* isolates from cassava and cacao were included in the ITS + LSU MLGs analysis, the robustness of the phylogenetic clustering remained high, with 100% bootstrap support across the NJ, MP, and ML for the *R. theobromae* clade (**Figure 5b**). A distinct subclade differentiated between cassava and cacao isolates, with bootstrap values of 66%, 68%, and 83% for cassava isolates and 86%, 83%, and 94% for cacao isolates under the NJ, MP, and ML methods, respectively (**Figure 5b**). The isolates used for this comparison and the respective GenBank accession numbers are listed in **Table 4**.

**Table 4:** Description of the *Rhizoctonia solani* and *R. theobromae* isolates used in the multilocus phylogenetic analysis (MLG) based on the ITS, LSU, and *atp6* regions, with collection details and GenBank accession numbers.

Isolate	Host	Geographic origin	Other name(s)	ITS	LSU	*atp6*
CBS 208.84	Bean	Japan	*T.sasakii*	DQ279038	KP171642	DQ301680
SA1-1	Soybean	Japan	*T. cucumeris*	KP171635	KP171643	DQ301602
CBS 970.96	Potato	USA, Alaska	*T. cucumeris*	DQ279005	KP171648	DQ301622
LAO1	Cassava	Lao PDR	*C. theobromae*	JBBBZN000000000		
DCWBS	Cassava	Brazil	*C. theobromae*	PQ192611	PV639453	PV247128
DCWBP	Cassava	Brazil	*C. theobromae*	PQ192612	PV639452	PV247129
South Sulawesi 1	Cocoa	Indonesia	*C. theobromae*	KU255725	KU319575	–
South Sulawesi 2	Cocoa	Indonesia	*C. theobromae*	KU255724	KU319576	–

The nucleotide identity of over 1, 000 contigs generated for DWBS and DWBP ranged from 95.7% to 100% when compared with the R*. theobromae* LAO1 reference genome (GCA_037974915.1) (**Table 4**). These contigs were obtained through *de novo* assembly using hybrid Illumina and Nanopore sequencing (ENA sample number: ERA33127699). This high sequence similarity supports the identification of the fungus associated with CWBD in Brazil as *R. theobromae*.

### 3.4 PCR-based detection

To further support the hypothesis that *Rhizoctonia theobromae* is associated with the epidemic outbreak in the state of Amapá, Brazil, PCR analyses were performed using the primer set CWBD-CIAT-F2/R2 described by Leiva et al. (2023), targeting the coding region of a putative Ca^2+^/calmodulin-dependent protein kinase (CaMK) gene (GenBank accession no. KAB5596398).

Among 50 cassava samples displaying disease symptoms, 37 tested positive for *R. theobromae*, indicating a strong association between pathogen detection and symptom expression. Based on the sampled dataset, the apparent diagnostic sensitivity among symptomatic plants was 74% (37/50), while specificity relative to asymptomatic field plants was 93% (14/15 negative). No false positives were detected among 40 healthy control plants collected from the Germplasm Bank in Cruz das Almas, Brazil (approximately 2, 500 km from the outbreak region), indicating high analytical specificity under the tested conditions (**Table 5**). These results support an association between *R. theobromae* and CWBD in affected fields.

**Table 5:** PCR analysis of field samples collected on the indigenous villages that are suffering with the outbreak (leaves, petioles and stem from plants with and without disease symptoms) showed a strong association of *Rhizoctonia theobromae* (*Ceratobasidium theobromae*) with CWBD.

Phenotype	Number of samples	PCR (−)	PCR (+)
Asymptomatic	15	14	1
Diseased	50	13	37
Healthy*	40	40	0

As means of comparison, plants randomly collected from the Embrapa Cassava Germplasm Bank expressing symptoms of anthracnose, frogskin disease and oversprouting were included.

*Plants collected from the Cassava Germplasm Bank in Cruz das Almas, Brazil, more than 2.500 km from the diseased fields, were considered healthy plants for *R. theobromae* infection.

DNA extracted from field samples consisted predominantly of cassava host DNA together with DNA from multiple microorganisms present in plant tissues. Consequently, the relative proportion of *Rhizoctonia theobromae* DNA represented only a small fraction of the total DNA recovered from infected plants. This effect was particularly evident in leaf tissues, where pathogen biomass appeared lower than in vascular tissues. Under these conditions, the low relative abundance of fungal DNA in total plant extracts reduced PCR detection efficiency and resulted in some symptomatic samples testing negative.

### 3.5 *Rhizoctonia theobromae* genetic diversity analysis

The putative pathogenicity-associated gene CAMK/CAMKL (~1, 100 bp), previously used for species-specific detection of *R. theobromae*, was also employed for population analysis to provide insight into the genetic diversity of the fungus in Brazil and its relationship with Asian *R. theobromae* isolates associated with CWBD. Eighteen high-quality CAMK/CMKL consensus sequences were retrieved from the samples and used for genetic diversity analysis (**Table 6**).

**Table 6:** Summary of cassava isolates used in the study of the diversity of putative Ca^2+^/calmodulin-dependent protein kinase genes (CAMK/CAMKL).

Sample	^1^Hap.	Cultivar	Year	Country	Location	Tissue	GenBank
AP-2204	H1	Bâton-Uaçá	2023	Brazil	Ariramba	Stem	PQ202677
AP-2207	H1	Bâton-Ló	2023	Brazil	Ariramba	Stem	PQ202678
AP-2303	H1	Tête-Bleu	2023	Brazil	Galibi	Stem	PQ202679
AP-2305	H1	Tête-Bleu	2023	Brazil	Galibi	Stem	PQ202680
AP-2351	H1	Bolinha	2023	Brazil	Galibi	Petiole	PQ202682
AP-2353	H1	Tête-Bleu	2023	Brazil	Galibi	Petiole	PQ202683
AP-2602	H1	Kawa’wa	2023	Brazil	Ahumã	Petiole	PQ202684
AP-2616	H1	Kalixá	2023	Brazil	Ahumã	Petiole	PQ202685
AP-2617	H1	Kawa’wa	2023	Brazil	Ahumã	Stem	PQ202686
AP-2663	H1	Camarão	2023	Brazil	Ahumã	Stem	PQ202687
AP-2706	H1	Tucumã	2023	Brazil	Anawerá	Leaf	PQ202688
AP-2711	H1	Tucumã	2023	Brazil	Anawerá	Stem	PQ202689
AP-2713	H1	Xingú	2023	Brazil	Anawerá	Leaf	PQ202690
AP-2719	H1	Xingú	2023	Brazil	Anawerá	Stem	PQ202691
AP-2802	H1	Manteiga	2023	Brazil	Yanawaka	Stem	PQ202692
AP-2807	H1	Manteiga	2023	Brazil	Yanawaka	Petiole	PQ202693
AP-2809	H1	Pretinha	2023	Brazil	Yanawaka	Petiole	PQ202694
AP-2812	H1	Manteiga	2023	Brazil	Yanawaka	Stem	PQ202695
YBa-1	H3	KM 94	2012	Vietnam	Yen Bai	Stem	OQ863061
YBa-2	H4	KM 94	2012	Vietnam	Yen Bai	Stem	OQ863062
YBa-3	H6	KM 94	2012	Vietnam	Yen Bai	Stem	OQ863063
YBa-4	H1	KM 94	2012	Vietnam	Yen Bai	Stem	OQ863064
YBa-5	H2	KM 94	2012	Vietnam	Yen Bai	Stem	OQ863065
HBi-1	H3	SC 205	2012	Vietnam	Hoa Binh	petiole	OQ863066
HBi-2	H3	SC 205	2012	Vietnam	Hoa Binh	petiole	OQ863067
DNa-1	H3	KM 94	2012	Vietnam	Dong Nai	Stem	OQ863068
DNa-2	H3	KM 94	2012	Vietnam	Dong Nai	petiole	OQ863069
DNa-3	H1	KM 94	2012	Vietnam	Dong Nai	Stem	OQ863070
Kra-1	H1	NA	2013	Cambodia	Kratie	Root	OQ863071
KCh-1	H3	NA	2013	Cambodia	Kampong Chan	Root	OQ863072
PVe-1	H3	NA	2013	Cambodia	Prey Veng	Root	OQ863073
TNi-1	H3	NA	2013	Vietnam	Tay Ninh	Root	OQ863074
TNi-2	H9	NA	2013	Vietnam	Tay Ninh	Root	OQ863075
Min-1	H9	NA	2017	Philippines	Mindanao	NA	OQ863076
Min-2	H8	NA	2017	Philippines	Mindanao	NA	OQ863077
Min-3	H10	NA	2017	Philippines	Mindanao	NA	OQ863078
Ray-1	H10	HB 60	2017	Thailand	Rayong	Root	OQ863079
Ray-2	H10	HB80	2017	Thailand	Rayong	Root	OQ863080
Ray-3	H10	^2^Ray.7	2017	Thailand	Rayong	Root	OQ863081
Ray-4	H8	Ray.86-13	2017	Thailand	Rayong	Root	OQ863082
Sal-1	H1	NA	2020	Lao PDR	Salavan	Leaf	OQ863083
Sal-2	H3	NA	2020	Lao PDR	Salavan	Leaf	OQ863084
Vie-1	H7	NA	2022	Lao PDR	Vientiane	Stem	OQ863085
Vie-3	H1	KU50	2023	Lao PDR	Vientiane	Stem	OQ863086
Vie-4	H5	Ray.11	2023	Lao PDR	Vientiane	Stem	OQ863087

The table includes information on sample identification, haplotype, cultivar, year of collection, country, specific location, tissue type, and corresponding GenBank accession numbers for isolates from various Asian countries and Brazil.

^1^Hap., Haplotype; ^2^Ray., Rayong.

Genetic analysis of *R. theobromae* based on the CAMK/CAMKL locus revealed marked differences in genetic diversity across regions. In Brazil, fungal isolates exhibited very low genetic diversity, with a Shannon diversity index of 0.12 and nucleotide diversity (π) of 0.001, suggesting limited variation within this dataset. Haplotype diversity in Brazil was 0.0, indicating the presence of a single detected haplotype. In contrast, Philippine isolates displayed higher diversity values (Shannon index = 1.56; π = 0.025; haplotype diversity = 0.85). Because this analysis is based on a single locus, these results reflect comparative genetic diversity patterns rather than definitive population structure.

**Figure 6:**
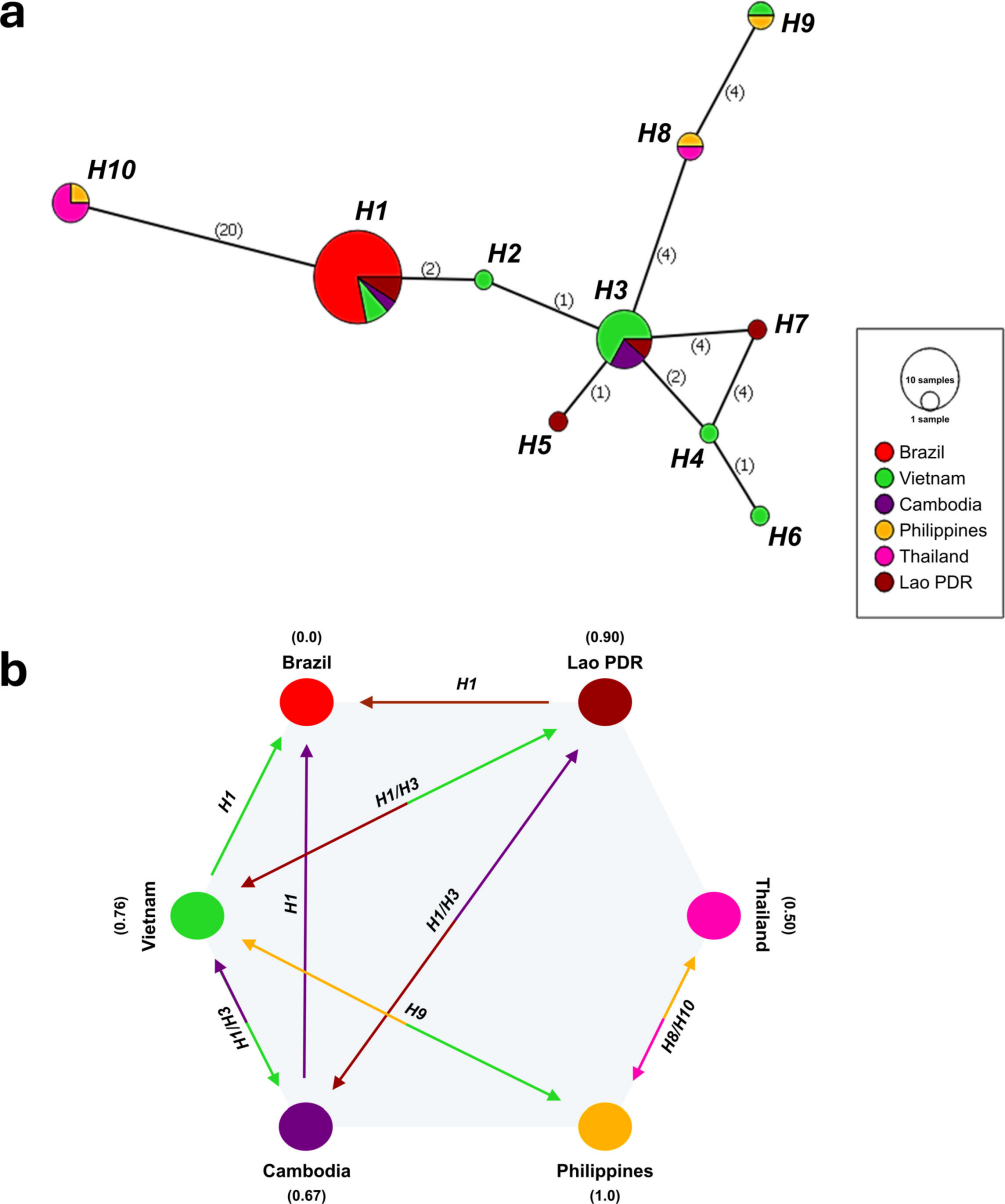
Diversity analysis of Brazilian and Asian *Rhizoctonia theobromae* isolates. **(A)** haplotype network of *Rhizoctonia theobromae* isolates based putative Ca2+/calmodulin-dependent protein kinase gene (CAMK/CAMKL). The network shows the number of individuals for each haplotype (circle size) and the population of origin (color). The numbers in parentheses next to the branches indicate the number of mutations that separate the different haplotypes. **(B)** diagram illustrating the sharing of haplotypes between the five Asian populations and Brazil. Values ​​in parentheses next to the names indicate the haplotype diversity (Hd) estimated for the population.

A high pairwise F_ST_ value (0.68) was observed between regional datasets, and the estimated migration rate (Nm = 0.5) was calculated based on this locus. However, because these metrics were derived from a single and potentially conserved gene, they should be interpreted cautiously. These values reflect differentiation at the CAMK/CAMKL locus and do not constitute definitive evidence of broader population genetic structure. The high concentration of individuals in the H1 haplotype, particularly in Brazil, may indicate a common ancestor or founder population (**Figure 6A**). The H1 haplotype is shared by Vietnam, Cambodia, and Laos, suggesting genetic connections or a common ancestor. This central haplotype is connected to other less frequent haplotypes, such as H2, H3, H4, and H5, which are found in more restricted populations.

The H10 haplotype, which is distant from the others, suggests a greater evolutionary divergence or an older founding event. Haplotype diversity was highest in the Philippines and, to a lesser extent, in Brazil and Laos, whereas Thailand showed the lowest diversity. The H3 haplotype is shared between Vietnam and Cambodia, indicating their genetic proximity. The connection between Thailand and the Philippines for the H8/H10 haplotypes suggests genetic sharing, possibly due to spore dispersal and/or transit of infected plants (**Figure 6B**).

## 4 Discussion

The outbreak of a new cassava disease in indigenous villages in Oiapoque, Amapá, Brazil, exhibits symptoms consistent with Cassava Witches’ Broom Disease (CWBD), including stunted growth, proliferation of weak shoots, broom-like structures on stems, chlorosis, wilting, leaf senescence, and apical dieback. These symptoms closely correspond with previous descriptions of witches’ broom disease in Brazil (Silberschmidt and Campos, 1944; Flôres et al., 2013), although CWBD is further distinguished by a characteristic rosette-like broom formation and vascular necrosis, leading to plant wilting.

Metagenomic analyses of diseased cassava samples revealed consistent microbial communities across sites, with fungal genera such as *Rhizoctonia theobromae*, *Aplosporella*, *Chrysoporthe*, and *Hebeloma* detected exclusively in symptomatic plants within the surveyed areas. Notably, *R. theobromae* was the dominant or sole fungal taxon in many CWBD samples. While these additional genera were isolated and are currently being evaluated for their potential pathogenic role, their contribution to disease development remains under investigation. In contrast, *R. theobromae* showed a consistent association with CWBD symptoms across all affected sites. This aligns with recent reports identifying *R. theobromae* as an emerging cassava pathogen in Asia (Leiva et al., 2023) and its presence in French Guiana (Pardo et al., 2024). Our detection of *R. theobromae* in the Oiapoque region represents the first report of its occurrence in the Brazilian Amazon, expanding its known geographic distribution in the Americas.

Phylogenetic analysis clustered *R. theobromae* isolates from cassava in Brazil with other isolates from cassava and cacao in Asia. ITS-based phylogeny provides a robust framework for delineating taxa within the *Rhizoctonia* complex and reinforces previous findings (Guest and Keane, 2018) regarding the monophyly of the Ceratobasidiaceae family. Multilocus genotyping (MLG) revealed a high genetic similarity between the Brazilian isolates and the *C. theobromae* LAO1 reference genome, further supporting the association of *R. theobromae* with CWBD in the region. Phylogenetic resolution using both *R. solani* and *R. theobromae* sequences revealed distinct subclades for cassava and cacao isolates, indicating possible host specialization, with implications for disease epidemiology and control.

*Ceratobasidium theobromae* has been implicated not only in CWBD but also in vascular streak dieback (VSD) of cacao (Pardo et al., 2024; Leiva et al., 2023; Gil-Ordóñez et al., 2024; Guest and Keane, 2018). Taxonomic complexity remains, as the teleomorphic state is classified as *Ceratobasidium*, whereas the anamorphic state is typically named *Rhizoctonia*. Some studies have also placed taxa related to the genus *Thanatephorus*. This overlapping classification highlights the importance of molecular phylogenetic tools in resolving such taxonomic ambiguities. Following current taxonomic revisions (Oberwinkler et al., 2013; O’Donnell et al., 2025), the accepted name *Rhizoctonia theobromae* is used throughout this manuscript.

PCR-based detection indicated an association between symptom expression and the presence of *R. theobromae*, supporting its potential epidemiological relevance. The higher detection rates observed in vascular tissues, particularly stems (93%) and petioles (95%), support the hypothesis that the pathogen preferentially colonizes the vascular system, consistent with the necrosis and wilting symptoms observed in affected plants. Occasional negative PCR results in symptomatic samples are likely explained by low pathogen DNA concentrations in total plant extracts, particularly in leaf tissues where fungal biomass is limited relative to host DNA. Moreover, the high sequence identity of the CAMK locus among isolates from Brazil and Asia suggests a shared genetic background. CaMK proteins are known to regulate fungal growth and pathogenicity-related processes in several plant-pathogenic fungi. Although functional characterization of the CAMK locus in *R. theobromae* has not yet been experimentally demonstrated, this locus has been previously validated as a species-specific molecular marker (Leiva et al., 2023; Pardo et al., 2024; Gil-Ordóñez et al., 2024), supporting its relevance for detection and comparative epidemiological analyses.

By integrating symptomology, high-throughput sequencing, and molecular diagnostics, this study provides evidence of a strong association between *R. theobromae* and CWBD in Amapá, Brazil. This is the first confirmed report of this pathogen in Brazil, marking a significant phytosanitary threat to both food security and the genetic diversity of local cassava landraces cultivated by indigenous communities. The pathogen may be spread via infected planting material and infested soil, with airborne basidiospores being a plausible, although yet unconfirmed, source of primary infection.

Given these findings, urgent mitigation efforts are required to address this issue. These include strict quarantine measures, the use of certified clean planting material, surveillance of cultivation zones, and risk communication targeting farmers and communities in affected areas. Public awareness campaigns and monitoring of crop movement across regions are essential to contain the spread of the disease.

*Rhizoctonia theobromae* (syn. *Ceratobasidium theobromae*, *Oncobasidium theobromae*, and *Thanatephorus theobromae*) have recently been designated as quarantine pests by Brazil’s Ministry of Agriculture and Livestock (MAPA), with notifications registered under SEI: 21157.001205/2024-84. Independent laboratory confirmation (files N° 03575/24-GO) triggered the implementation of quarantine protocols (MAPA, 2024). The detection of this pathogen in Amapá threatens not only cassava production but also cacao (*Theobroma cacao*) and potentially other crops of economic importance, such as *T. grandiflorum* (“cupuaçú”).

Although global cassava disease management has focused predominantly on viral threats, such as Cassava Brown Streak Disease and Cassava Mosaic Disease (Uke et al., 2022; Hareesh et al., 2023), the emergence of CWBD calls for broader disease surveillance and horizon-scanning frameworks. Early detection and rapid response systems must be enhanced to prevent the establishment and spread of novel, previously unrecognized pathogens such as *R. theobromae*.

### 4.1 Conclusion

Cassava Witches’ Broom Disease (CWBD) in the Oiapoque region of Brazil is characterized by stunted growth, abnormal shoot proliferation, and distinct broom-like structures, with vascular necrosis consistently associated with *R. theobromae*. Metagenomic and phylogenetic analyses support an association between *Rhizoctonia theobromae* and CWBD in Brazil, consistent with reports from Southeast Asia. The low genetic variability observed among Brazilian isolates, including the detection of a single haplotype, is consistent with a possible recent introduction scenario, although this interpretation remains preliminary. However, the greater genetic diversity reported in Asian populations may partly reflect broader geographic and temporal sampling in that region. Therefore, while our data support a shared lineage between Brazilian and Asian isolates, additional multilocus and population-level analyses are necessary to clarify the evolutionary history and dispersal patterns of this pathogen. The inability to reproduce the full disease phenotype under controlled conditions indicates that further studies are required to establish a definitive causal role.

## Author’s note

All sampling and genetic analyses were conducted under SISGEN registration AFB8348, in compliance with Brazilian Law 13.123/2015 and Decree 8.772/2016.

## Data Availability

The datasets presented in this study can be found in online repositories. The names of the repository/repositories and accession number(s) can be found below: https://www.ncbi.nlm.nih.gov/genbank/, PQ192611, PV639453, PV247128, PQ192612, PV639452, PV247129 https://www.ebi.ac.uk/ena, ERA33127699.
